# Diurnal and nutritional adjustments of intracellular Ca^2+^ release channels and Ca^2+^ ATPases associated with restricted feeding schedules in the rat liver

**DOI:** 10.1186/1740-3391-11-8

**Published:** 2013-08-20

**Authors:** Adrián Báez-Ruiz, Karina Cázares-Gómez, Olivia Vázquez-Martínez, Raúl Aguilar-Roblero, Mauricio Díaz-Muñoz

**Affiliations:** 1 Departamento de Neurobiología Celular y Molecular, Instituto de Neurobiología, Campus UNAM-Juriquilla, Apdo. postal 1–1141, Querétaro 76001, México; 2 Departamento de Neurociencias, Instituto de Fisiología Celular, Universidad Nacional Autónoma de México, Mexico City, MÉXICO; 3 Departamento de Neurobiología Molecular y Celular, Instituto de Neurobiología, UNAM-Juriquilla, Boulevard Juriquilla #3001, Apdo. Postal 1–1141, Querétaro, QRO 76230, México

**Keywords:** Inositol 1,4,5 trisphosphate receptor, Ryanodine receptor, SERCA, PMCA, Restricted feeding, Food entrained oscillator, Zonal distribution

## Abstract

**Background:**

Intracellular calcium is a biochemical messenger that regulates part of the metabolic adaptations in the daily fed-fast cycle. The aim of this study was to characterize the 24-h variations of the liver ryanodine and IP_3_ receptors (RyR and IP_3_R) as well as of the endoplasmic-reticulum and plasma-membrane Ca^2+^-ATPases (SERCA and PMCA) in daytime restricted feeding protocol.

**Methods:**

A biochemical and immunohistochemical approach was implemented in this study: specific ligand-binding for RyR and IP_3_R, enzymatic activity (SERCA and PMCA), and protein levels and zonational hepatic-distribution were determined by immunoblot and immunohistochemistry respectively under conditions of fasting, feeding, and temporal food-restriction.

**Results:**

Binding assays and immunoblots for IP_3_R1 and 2 showed a peak at the light/dark transition in the *ad-libitum* (AL) group, whereas in the restricted-feeding (RF) group the peak shifted towards the food-access time. In the case of RyR binding experiments, both AL and RF groups showed a modest elevation during the dark period, with the RF rats exhibiting increased binding in response to feeding. The AL group showed 24-h rhythmicity in SERCA level; in contrast, RF group showed a pronounced amplitude elevation and a peak phase-shift during the light-period in SERCA level and activity. The activity of PMCA was constant along day in both groups; PMCA1 levels showed a 24-h rhythmicity in the RF rats (with a peak in the light period), meanwhile PMCA4 protein levels showed rhythmicity in both groups. The fasted condition promoted an increase in IP_3_R binding and protein level; re-feeding increased the amount of RyR; neither the activity nor expression of SERCA and PMCA protein was affected by fasting–re-feeding conditions. Histochemical experiments showed that the distribution of the Ca^2+^-handling proteins, between periportal and pericentral zones of the liver, varied with the time of day and the feeding protocol.

**Conclusions:**

Our findings show that RF influences mainly the phase and amplitude of hepatic IP_3_R and SERCA rhythms as well as discrete zonational distribution for RyR, IP_3_Rs, SERCA, and PMCA within the liver acinus, suggesting that intracellular calcium dynamics could be part of the rheostatic adaptation of the liver due to diurnal meal entrainment/food entrained oscillator expression.

## Background

The liver is the primary metabolic processor of nutrients, and hence, it is deeply involved in digestive physiology. Feeding behavior is a complex set of events controlled by neural and hormonal communication between structures of the central nervous system (mainly hypothalamic structures such as the arcuate, lateral, ventromedial and paraventricular nuclei) and a variety of organs related to nutrient handling in the digestive tract [1]. Given that in nature the food sometimes is scarce, organisms have developed strategies to optimize the finding of food, the processing of nutrients, and the assimilation of biomolecules precisely when mealtime result a predictable event. Part of these adaptations is a timing system that underlies the events involved in the circadian rhythmicity. It is in this context that, in conditions of restricted access to food, the expression of an oscillator synchronized by food (FEO) is observed, which is an circadian alternative to the role played by the suprachiasmatic nuclei [2,3]. When animals are under a restricted feeding schedule over a period of several weeks, they display a behavior known as food anticipatory activity (FAA), an arousal behavior that precedes the availability of food. This FAA behavior is associated with the FEO [4].

The anatomical location of the FEO is still undetermined. However, the liver is likely playing a role in FEO physiology since it acts as a time-driven metabolic integrator of nutrients and has been associated with the control of the hunger-satiety cycle [5-7]. For example, a decrease in liver ATP triggers feeding behavior as well as an increase in the hepatic cytosolic Ca^2+^ concentration after administration of the metabolic inhibitor fructose analogue 2,5-anhydro-D-mannitol (2,5-AM) [8]. Upon re-feeding, rats display compensatory hyperphagia with a concomitant increase in ATP synthesis [9]. Many parameters related to hepatic physiology display circadian rhythmicity driven by the so-called clock genes, but they are also entrained by food availability [10]. In this context, the liver acts as a peripheral oscillator with the capacity to exhibit circadian rhythms in metabolic and physiological activities, eliciting an extremely rapid response when a restricted feeding (RF) protocol is applied [11].

During FAA, changes occur in: 1) the expression of ~80 hepatic genes related to biochemical reactions [12], 2) the cytoplasmic and mitochondrial redox state, which becomes oxidized [13], 3) ATP levels and mitochondrial respiration, which show an elevation [14], and 5) glycogenolytic activity, which is reduced in comparison with 24-h-fasted rats [15]. In addition, RF promotes lipid mobilization from adipose tissue followed by an increased catabolism of fatty acids within the liver [16-18]. The metabolic pattern found in rats entrained by daily restricted-food access/expressing the FEO is different from the one displayed by rats fed *ad-libitum* and under a 24 h of fasting, suggesting that during the RF protocol, hepatic physiology adopts a novel regulatory condition known as rheostasis or allostasis, a term meaning stability through regulated changes [19].

In the liver, intracellular calcium modulates glucose metabolism (glycogenolytic and glyconeogenic activities), protein folding, mitochondrial function, gene transcription, apoptosis, cell proliferation, and bile secretion [20]. Calcium modulates all these processes based on the temporal and spatial transients that function as a metabolic and transcriptional control code [21]. Intracellular calcium dynamics involves the coordinated action of a variety of proteins responsible for calcium mobilization outside of and within the cytosolic space. The liver expresses the 2 principal intracellular, calcium-release channels: the inositol 1,4,5-trisphosphate receptor (IP_3_R) (types 1 and 2) [22,23] and the ryanodine receptor type 1 (RyR), detected as a truncated but functional channel-protein [24]. The hepatic metabolic pumps that extrude cytosolic calcium are the sarco/endoplasmatic reticulum calcium ATPase (SERCA) splicing isoform 2b and the plasmatic membrane calcium ATPase (PMCA) type 1 and 4, but isoform 2 is also expressed at a low level [25].

Liver acinus shows two distinctive zones in which hepatocytes exhibit biochemical heterogeneity depending on the arrival of oxygen and nutrients. Hepatocytes next to portal vein are named periportal (PP) and show higher rates of glyconeogenesis, urea synthesis, bile formation, and lipid catabolic activity. Hepatocytes near the central vein are called pericentral (PC), and show mainly glycolysis, glycogenolysis, a high content of cytochromes P-450, and detoxification activities [26]. It was reported that IP_3_R is heterogeneously distributed along the hepatic acinus, being more abundant in the PP than in the PC zone [27]. No reports exist regarding liver zonational distribution for other calcium-handling proteins.

Liver calcium signaling responds to the energy status and hence to the feeding condition. Hepatocytes from fasting rats show significantly higher cytosolic calcium levels [28]. In addition, as mentioned previously [8], rats treated with 2,5-AM showed a significant reduction of hepatic ATP levels and a concomitant elevated intracellular calcium response. Our hypothesis is that the biochemical properties and zonal location of receptor-channels and ATPases handling intracellular calcium in the liver will be regulated by the timing system and will be responsive to a feeding protocol of food access restricted to 2 h during daytime. Hence, the aim of this report was to explore the influence of restricted feeding and the associated FEO expression on the daily variations of the main hepatic intracellular calcium-handling proteins: IP_3_Rs, RyR, SERCA, and PMCAs.

## Methods

### Materials

Antibodies against PER1 (sc-7724), IP_3_R1(sc-6093) and IP_3_R2 (sc-7278), SERCA2 (sc-8094), PMCA1 (sc-16488) and PMCA4 (sc-22080), and Actin as well as alkaline phosphatase (AP)-conjugated rabbit anti-goat and goat anti-mouse secondary antibodies were obtained from Santa Cruz Biotechnology (Santa Cruz, CA, USA), and the Ryanodine Receptor (ab9078) was from Millipore (MA, USA). [^3^H]-IP_3_ and [^3^H]-ryanodine were purchased from New England Nuclear (NEN, MA, USA). (1,4,5)-Inositol trisphosphate and ryanodine were from Calbiochem (CA, USA). Protease inhibitors, Stains all reactive and all other chemicals were obtained from Sigma (MO, USA), and Western blot equipment and reagents were from Bio-Rad (CA, USA).

### Animals and housing

Adult male Wistar rats weighing 200 ± 20 g (11–12 weeks old) at the beginning of the experiment were maintained under a 12 h:12 h light–dark cycle (light on at 08:00 h) at constant temperature (22 ± 1°C). Rats were kept in separate groups of 4 in transparent acrylic cages (40 × 50 × 20 cm), with free access to water and balanced Purina Chow meal except during food restriction, fasting, or re-feeding conditions. Illumination during the light period was obtained from 40 W fluorescent bulbs that generated 120 lux at the cage lid. All experimental procedures were conducted in accordance with our Institutional Guide for Care and Use of Animal Experimentation (Universidad Nacional Autónoma de México) and in agreement to international ethical standards [29].

### Experimental design

Rats were randomly assigned to 4 groups: 1) rats fed *ad libitum* (AL) for 3 weeks; 2) rats exposed to a daily restricted feeding schedule (RF) with access to food only between ZT4 to ZT6 (ZT0 is the time of lights on) for 3 consecutive weeks; 3) rats under 21 h of food deprivation (Fasted) starting at ZT6, and 4) rats that were fasted for 22 h (starting at ZT6 on the first day) and re-fed (Refed) for 2 h (from ZT4 to ZT 6 on the second day). At the end of the third week, rats from the AL and RF groups were sacrificed at 3-h intervals to complete a 24-h, day-night cycle (from ZT0 to ZT21). The Fasted and Refed group were sacrificed at ZT3 and ZT6, respectively. RF group was used to characterize the effect of temporal restricted feeding on calcium-handling proteins 24 h profiles, meanwhile Fasted and Refed groups were used as acute feeding condition controls for the RF groups at ZT3 (before food access and during FAA) and ZT6 (after feeding) respectively [18].

### Corticosterone determination

Samples were collected from trunk blood and centrifuged at 4,000 g for 15 min at 4°C. Plasma was then stored at −80°C for subsequent measurements of corticosterone. Corticosterone concentrations were measured in duplicate using a commercial ELISA kit (Assay Designs, MI, USA).

### Subcellular fractionation

Hepatic tissue was fractionated as reported by [30]. The liver was removed (≈5 g), immediately placed in ice-cold homogenization buffer (HB, 1:10 w/v), and disrupted with a Potter-Elvehjam teflon-glass homogenizer (40 rpm for 15–20 s). The HB contained: 225 mM sucrose, 0.3 mM EGTA, 10 mM Tris/HCl (pH 7.4), and 1 mM DTT, supplemented with a mixture of protease inhibitors (0.1 mM PMSF, 0.1 mM benzamidine, 10 μM pepstatin A, 1 μg/ml aprotinin, 1 μg/ml *o*-phenanthroline, and 10 μg/ml leupeptin). The liver homogenate was centrifuged at 1,000 g for 15 min (in a Sorvall SS34 centrifuge), and the resulting supernatant was decanted. The supernatant was centrifuged 2 times at 7,700 g for 15 min to precipitate the mitochondrial fraction. The resultant supernatant was ultracentrifuged (Beckman 70Ti rotor) at 100,000 g for 60 min. The pellet, corresponding to the endoplasmic reticulum fraction (ER), and the supernatant, corresponding to the cytosol fraction, were collected, aliquoted, and kept at −70°C. The plasma membrane fraction (PM) was obtained from the first 7700 × g pellet, as described by [31]. The pellet was resuspended in 20 ml of 250 mM sucrose - 10 mM Tris/HCl (pH 7.5) solution, then mixed with 2.6 ml of Percoll (1.13 g/ml density) and 0.4 ml of 2 M sucrose, placed at the top of a Percoll gradient, and centrifuged at 35,000 g for 20 min to yield the crude PM fraction in the supernatant. This fraction was carefully recovered (~15 ml), layered on 5 ml of the same buffer previously described, with the addition of sucrose/Tris buffer containing 1.3 M CaCl_2_, and centrifuged at 37,000 g for 25 min; the PM fraction was obtained from the middle of the gradient, separated from other cellular components. The total membrane fraction from skeletal muscle was processed as described by [32] and was used as a tissue control as refereed forward. Subcellular fractions were resuspended in HB and stored in aliquots at −70°C until further use. Protein concentration was determined by the Lowry method [33] using bovine serum albumin (BSA) as standard.

### Marker enzymes

Glucose-6-phosphatase activity (EC 1.1.1.49) was assayed as described by [34] and used as an endoplasmic reticulum (ER) marker; 5´nucleotidase activity (EC 3.1.3.5) was determined following the method of [35], and was used to evaluate the purity of the plasma membrane fraction (PM). Both enzymes were determined first in the total homogenate and later in the isolated fractions.

### [^3^H]-IP_3_ Binding

The [^3^H]-IP_3_ binding assay was performed as reported by Furiuchi et al. [36] with some modifications: 100–300 μg of microsomal and PM fractions were incubated in triplicate for 30 min in 120 μl of a solution containing 25 mM Tris/HCl (pH 8.0), 5 mM NaHCO_3_, 1 mM EDTA, 0.25 mM DTT, and 0.5 to 100 nM of [^3^H]-IP_3._ Non-specific binding was measured as the radioactivity not displaced by non-radioactive 10 μM IP_3_. Each sample was washed 5 times with 5 ml of a cold buffer containing 25 mM Tris/HCl (pH 8.0), 5 mM NaHCO_3_, and 1 mM EDTA. Filters were counted in 10 ml tritosol [37] in a LS6500 Beckman multi-purpose scintillation counting system.

### [^3^H]-Ryanodine binding

[^3^H]-Ryanodine binding was evaluated as described by Hamilton et al. [38]. Briefly, 400 μg of microsomal protein was incubated in triplicate for 14–16 h with [^3^H]-ryanodine at concentrations ranging from 0.5 to 500 nM, in a final volume of 250 μl of incubating buffer: 300 mM KCl, 100 μM CaCl_2_, 100 μg/ml BSA, and 20 mM MOPS (pH 7.4) at room temperature. The assay was terminated by addition of 5 ml ice-cold 0.3 mM KCl and filtration on Whatman GF/F filters, followed by another 4 washes. Non-specific binding was defined as that not displaced by the addition of 10 μM of non-radioactive ryanodine. Radioactivity bound and binding parameters (Bmax and *Kd*) were determined as described previously for IP_3_R binding assay.

### Ca^2+^ATPase activity assays

ATPase activities were measured by a standard coupled enzymatic assay in which the rate of ATP hydrolysis was linked to NADH oxidation, and the optical density was recorded at 340 nm (ϵ = 6.22 mM^-1^.cm^-1^). ER and PM samples were used for SERCA and PMCA assays as described by Saborido et al. [39]. Aliquots (50 to 100 μg) of each fraction were incubated in buffer containing 25 mM MOPS (pH 7.4), 0.2 mM EGTA, 5 mM MgCl_2_, 100 mM KCl, 0.6 mM phospho*enol*pyruvate, 2.4 unit/ml pyruvate kinase, 10 unit/ml lactate dehydrogenase, 4 μM ionophore A23187, 0.27 mM NADH, and 1 mM CaCl_2_ for total activity (21 mM CaCl_2_ for basal activity) in a final volume of 1 ml. After pre-incubation of the assay mixture for 5 min at 37°C, the reaction was started by adding 1 mM ATP (final concentration). SERCA and PUMP Ca^2+^-ATPase activities were calculated as total activity minus the basal activity. Thapsigargin (1 μM) and Eosin (2 μM) were used as SERCA and PMCA inhibitors, respectively, in order to test the specificity of the measured activity of the two pumps [31].

### Western blot analysis

Cellular fractions were resuspended in SDS sample buffer in reducing conditions, as described by Laemli [40]. Proteins were separated by SDS-PAGE using 5% acrylamide for IP_3_R (type 1 and 2) and RyR, and 7.5% acrylamide for PER1, SERCA2, and PMCA (types 1 and 4). After electrophoresis, proteins were transferred to Protean nitro-cellulose membranes and blocked with PBS containing 0.1% Tween and 5% defatted milk for 1 h at room temperature. Membranes were incubated with the primary antibody overnight. All the antibodies were used at 1:500 dilutions, except that the antibody used to detect the skeletal muscle RyR protein as positive control not detected the hepatic RyR protein (even using other RyR antibodies from the companies Santa Cruz and Abcam). Then stains-all protocol was applied in order to detect the hepatic RyR protein using its characteristic very high molecular weight (more than 500 kDa) as a defining parameter [24]. After repeated washes with PBS-Tween buffer, membranes were incubated for 2 h with the appropriate alkaline phosphatase (AP)-conjugated secondary antibody at 1:5000 dilution, and the bands were visualized using the AP conjugate substrate kit (Bio-Rad, CA, USA) according to the manufacturer’s instructions. Membrane samples from cerebellum (IP_3_R1), kidney (IP_3_R2), skeletal muscle (RyR) and brain (SERCA2b and PMCA1-4 proteins) were used as positive controls. Blots were digitalized and analyzed with the Image J® software (version 1.38, USA).

### Immunohistochemistry

To determine the acinus (PP and PC) distribution of RyR, IP_3_R1 and 2, SERCA2, and PMCA1 and 4, slices of rat liver (10 μm thick) were incubated with specific antibodies. Immunohistochemistry was performed as reported by Clair et al. [41] using freshly isolated liver slices fixed in 4% (w/v) paraformaldehyde-PBS, pH 7.5 overnight, and cryo-preserved in 30% (w/v) sucrose for another overnight cycle. After permeabilization with 0.05% Triton-PBS for 10 min and blocking with PBS containing 5% de-fatted milk plus 0.1% BSA, slices were incubated overnight at 4°C with primary antibody at concentration suggested by the manufacturer. After rinsing in PBS 3 times, the liver tissue slices were incubated at room temperature for 1 h with a secondary antibody conjugated to FITC (fluorescein 5-isothiocyanate) supplied by Sigma (MO, USA). Actin related to apical membrane was stained with Rhodamine-conjugated Phalloidine in order to outline hepatic cells [42].

### Visualization and quantification of fluorescence

Liver slices were visualized using a CX31 Olympus microscope. Images were collected with a DP71 Olympus camera and visualized using Image-Pro Plus software (version 6.0, MD, USA). Throughout the study, standardized fluorescence was set in all proteins studied against their respective negative control (without specific primary antibody). Fluorescent intensity in PP and PC hepatocytes was quantified as described by Lahm et al. [43].

### Calculations and statistics

The results are expressed as mean ± SEM of at least 4 individual experimental observations. Statistical analysis was done using the Prism version 5.0 program (GraphPad software, USA). To detect possible time-condition differences, RF and Al groups were compared with a two-way ANOVA. Significance was estimated by the Tukey test with an α level set at 0.05. For the feeding condition controls, a Student´s t-test was used to detect significant differences between fasted vs re-fed, fasted vs RF ZT3, re-fed vs RF ZT6, and RF ZT3 vs RF ZT6. For chronobiological analysis, first a one-way ANOVA was performed in each group and then a 24-h period single-cosinor method was used as previously described [44]. For rhythmic interpretation of the results, the following parameters were considered: acrophase (time of peak value), MESOR (midline estimating statistic of rhythm), amplitude (half of the total variation of the rhythm), and rhythmicity, which corresponds to a p value (< 0.01) of an F test of fitting the original results to an expected sinusoidal curve with a 24-h period.

## Results

### Establishment of the FEO expression / restricted feeding protocol

**Figure 1:**
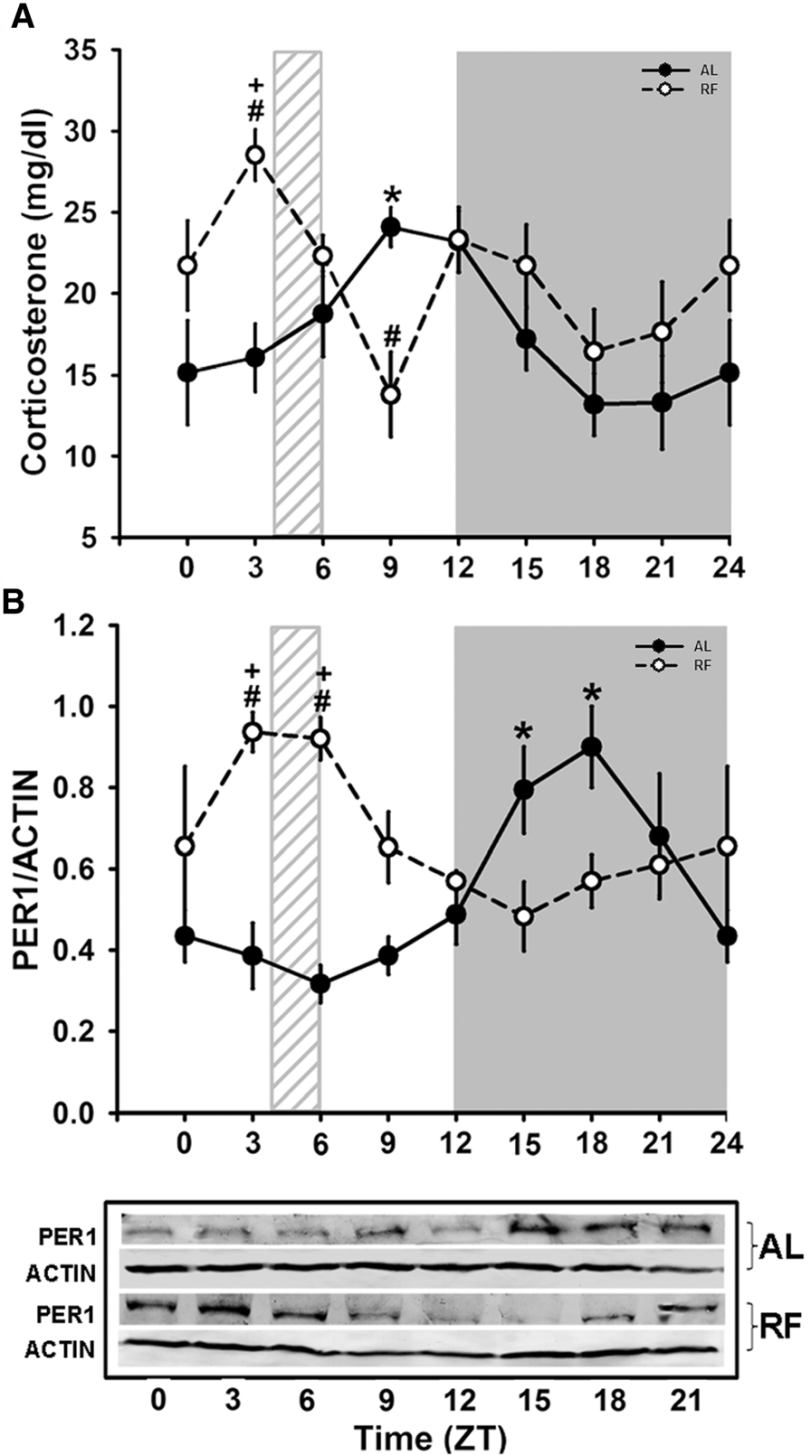
**Serum corticosterone and PER1 protein levels are entrained by food access.** Diurnal corticosterone levels in rats fed *ad libitum* (AL) and under RF are shown in panel **A.** Quantitative analysis of clock protein PER1 using 50 μg of cytosolic fraction from at least 4 individuals is represented in panel **B.** Each value was normalized using the housekeeping protein actin as reference, and a representative western blot for each condition is shown. Black circles correspond to AL and white circles to RF group. The light gray rectangle above the x-axis represents mealtime for the food restricted group (ZT4-ZT6); * (*p* < 0.05) means significant difference among AL group time points and **+** (*p* < 0.05) significant difference among RF group time points (1-way ANOVA). # (*p* < 0.05) significant difference between AL vs RF (2-way ANOVA).

To confirm the metabolic and physiological adaptations associated with the protocol for RF (food access only during 2 daytime hours) and the FEO expression, serum corticosterone levels and the presence of liver PER1 protein were determined over a 24-h cycle (Figure 1). It is well accepted that RF promotes a significant shift in the peaks of these 2 parameters during the time of the FAA, prior to food access [45,46]. Indeed, it is observed in Figure 1A that AL and RF rats showed an elevation of circulating corticosterone previous to the end of the light period when the animals are in the transition between the sleep and the awake periods (around 50% from the trough; AL group, *p < 0.009*, one-way ANOVA). But, in addition, the RF group showed another, even larger peak at ZT3 before feeding (100% increase with respect to the trough; RF group, *p < 0.004*, one-way ANOVA). A similar pattern was observed in the 24-h rhythm of PER1 (Figure 1B): the AL group showed a clear PER1 peak during the dark period (ZT15–Z21; *p < 0.009*, one-way ANOVA) when the animals are active, confirming previous reports [47]. In contrast, RF rats showed a shift of the PER1 peak towards the light period (ZT3-ZT6), which corresponds to the period immediately before and after feeding. According to the two-way ANOVA test, levels of corticosterone and PER1 protein from AL and RF groups (Figure 1) differed significantly, indicating a rhythmicity that was characterized by a Cosinor test. Corticosterone and PER1 daily rhythms from the AL group have significant 24-h rhythmicity (Table 1), but in the RF group these parameters exhibited a phase advance (of ~7 h and ~12 h, respectively). PER1, but not corticosterone, showed a significant 24-h rhythm (Table 1) in the RF group. This result is due to the biphasic pattern of daily corticosterone levels (one corresponding to light entrained before dark period and the other related to meal cue before food access time). Both results demonstrated that our protocol successfully induces hepatic entrainment due to daytime restricted feeding.

**Table 1:** Daily rhythm characteristics of plasma corticosterone and PER1 from rats under free access meal and restricted schedule determined by COSINOR test

	**Acrophase ZT (h)**		**Amplitude**		**MESOR**		**Rhythmicity ( *P < 0.05 *)**	
	**AL**	**RF**	**AL**	**RF**	**AL**	**RF**	**AL**	**RF**
**Corticosterone**	09:30 ± 00:26	02:15 ± 00:35*	3.13 ± 0.23	3.39 ± 0.30	17.6 ± 3.9	20.7 ± 4.3	0.005	0.6^NS^
**PER1 (WB)**	17:30 ± 00:19	05:06 ± 00:24*	0.27 ± 0.015	0.22 ± 0.011	0.55 ± 0.07	0.64 ± 0.06	0.002	0.04

The Acrophase, amplitude, and MESOR as well as the rhythmicity significance were determined as described in the Methods section. The values were determined from the diurnal profiles of these parameters. The units corresponding to amplitude and MESOR are mg/dl (corticosterone) and normalized units (PER1/ACTIN Western Blot). * (*p* < 0.05) significant between AL vs RF (Student´s t-test). Not significant 24 h rhythmicity was designed as NS.

### IP_3_R And RyR: ligand binding properties and protein expression during restricted feeding / FEO expression

**Figure 2:**
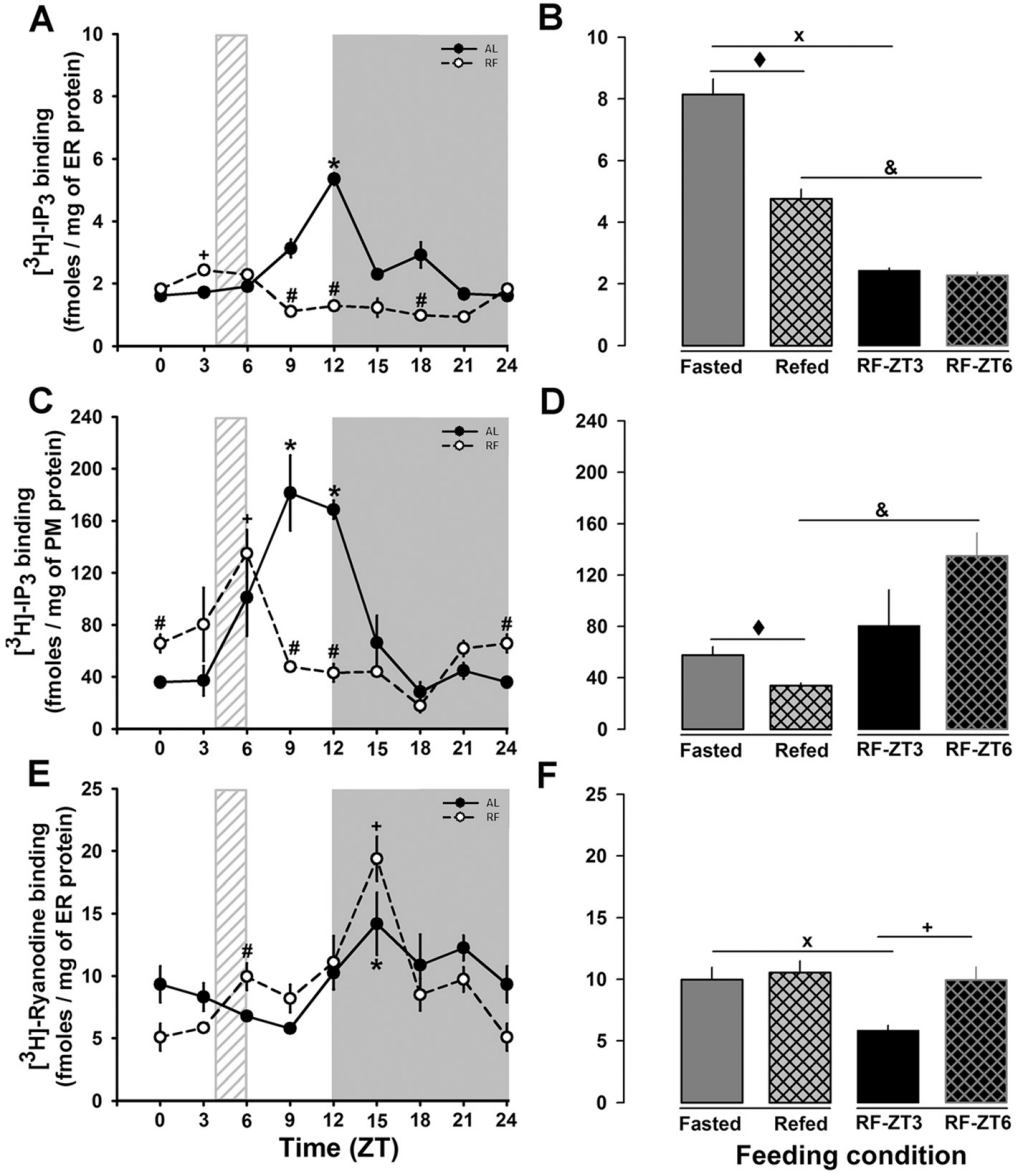
**Intracellular calcium-releasing channel binding assay.** Binding activity of IP_3_R and RyR was measured as described in Methods. Panels **A** and **C** show daily patterns of the binding activity calculated for the IP_3_R in the ER and PM fractions, respectively. Panels **B** and **D** compare the feeding condition groups (Fasted and Refed) for IP_3_R binding in the ER and PM fractions, respectively. Panel **E** shows the daily pattern of the RyR binding activity. Panel **F** compares feeding condition groups for RyR-binding activity. Black circles correspond to AL, and white circles to RF group. The light gray rectangle above x-axis indicates meal time for the food restricted group (ZT4-ZT6). Mean values for four separate experiments are shown. Each data point was carried out in triplicate. * (*p* < 0.05) significant difference between AL time points and **+** (*p* < 0.05) significant difference in the RF group between their time points (1-way ANOVA); # (*p* < 0.05) significant between AL vs RF (2-way ANOVA). ♦ (*p* < 0.05) significant difference between Fasted and Refed groups; x (*p* < 0.05) significant difference between Fasted and RF-ZT3; and & (*p* < 0.05) significant difference between Refed and RF-ZT6 (Student´s t-test).

Diurnal fluctuations of liver IP_3_R and RyR were determined in subcellular fractions. To characterize the hepatic endoplasmic reticulum (ER) and plasma membrane (PM) fractions, the specific activities of glucose-6-phosphatase (ER marker) and 5´nucleotidase (PM marker) were measured (data not shown). The recovery yield was similar in AL and RF groups at the different tested time (data not shown). The maximum contamination of PM in the ER fraction was 13%, whereas the ER contamination in the PM fraction was 18%. According to the one-way ANOVA test, the [^3^H]-IP_3_ binding in ER membranes of the AL group showed significant differences over the 24-h period (Figure 2A). Although the RF group showed significant differences at certain time points, there was a clear decrease in the amplitude of [^3^H]-IP_3_ binding rhythm. Both groups showed 24-h rhythmicity. In addition, the RF group showed an almost 50% reduction in amplitude and MESOR as well as a phase advance of 8 h with respect to the AL group (Table 2). Interestingly, [^3^H]-IP_3_ binding in the ER fraction of the Fasted group increase in comparison to the Refed group but also reach the highest value of all experimental groups. The same pattern occur in AL group since [^3^H]-IP_3_ binding decrease after rats start it activity period (Figure 2A). In contrast, the RF group did not show any difference between ZT3 (before feeding) and ZT6 (just after feeding), but both values were significantly lower than the corresponding control groups of feeding condition (Figure 2B). In the hepatic ER fraction, the IP_3_R type 1 is the main isoform, but type 2 is also present at lower levels [20]. Hence, the [^3^H]-IP_3_ binding was also measured in the PM fraction which has been suggested as an IP_3_R type 2-enriched subcellular site [42]; the data indicated significant differences among time points for both groups (p < 0.001, one-way ANOVA, Figure 2C) as well as between the AL and RF groups (p < 0.01, one-way ANOVA). These results are consistent with the differences shown in Table 2 regarding 24-h rhythmicity: RF promoted a 5-h advance, a 50% reduction of amplitude (relative to the AL group) and a ~25% decrease of MESOR. Regarding feeding condition control groups, a decrease in [^3^H]-IP_3_ binding was observed in the PM fraction of the Refed group (Figure 2D). However, the RF group again displayed a dissimilar pattern: At ZT6, RF rats showed a significant PM [^3^H]-IP_3_ binding elevation in comparison to the Refed group (Figure 2D).

**Table 2:** Characteristics of the diurnal rhythms of hepatic calcium-handling proteins from rats under free access meal and restricted schedule determined by COSINOR test

		**Acrophase ZT (h)**		**Amplitude**		**MESOR**		**Rhythmicity ( *P < 0.05 *)**	
		**AL**	**RF**	**AL**	**RF**	**AL**	**RF**	**AL**	**RF**
**Ca**^**2+ **^**releasing proteins**	**IP**_**3**_**R (ER Binding assay)**	12:20 *+* 00:21	04:02 *+* 00:20*	1.3 *+* 0.22	0.6 *+* 0.10*	2.6 + 0.44	1.5 + 0.21*	0.01	0.05
	**IP**_**3**_**R1 (WB)**	12:05 + 00:35	05:37 + 00:47*	0.28 + 0.04	0.16 + 0.03*	0.56 + 0.08	0.37 + 0.06*	0.03	0.04
	**IP**_**3**_**R (PM Binding assay)**	09:57 + 00:48	04:22 + 00:28*	42.8 + 10.3	36.3 + 6.1	82.9 + 21.7	61.9 + 12.3*	0.02	0.05
	**IP**_**3**_**R2 (WB)**	11:26 + 00:29	04:03 + 00:31*	0.29 + 0.034	0.25 + 0.036	0.54 + 0.07	0.31 + 0.07*	0.02	0.03
	**RyR (Binding assay)**	17:43 + 00:41	17:10 + 00:32	3.2 + 0.46	4.3 + 0.78	9.7 + 0.9	10.2 + 1.5	0.03	0.05
	**RyR (WB)**	17:50 + 00:50	17:30 + 00:58	0.29 + 0.033	0.22 + 0.052	0.59 + 0.06	0.62 + 0.10	0.03	0.3^NS^
**Ca**^**2+ **^**extruding proteins**	**SERCA (Activity)**	18:35 + 00:31	03:51 + 00:39*	5.36 + 0.9	20.9 + 2.4*	23.2 + 2.1	47.4 + 5.8*	0.2^NS^	0.004
	**SERCA2 (WB)**	16:48 + 00:29	02:55 + 00:24*	0.24 + 0.02	0.34 + 0.03*	0.49 + 0.04	0.66 + 0.07*	0.4^NS^	0.02
	**PMCA (Activity)**	21:48 + 01:09	23:44 + 01:07	6.2 + 1.2.	2.7 + 0.8*	19.8 + 2.3	21.6 + 1.4	0.2^NS^	0.5^NS^
	**PMCA1 (WB)**	01:35 + 0:59	09:15 + 00:45*	0.1 + 0.02	0.2 + 0.03	0.8 + 0.04	0.67 + 0.06	0.2^NS^	0.02
	**PMCA4 (WB)**	12:48 + 00:32	17:37 + 00:24*	0.24 + 0.04	0.3 + 0.05	0.66 + 0.08	0.53 + 1.0	0.06^NS^	0.04

The Acrophase, amplitude, and MESOR as well as the rhythmicity significance were determined as described in the methods section. Values were determined from the diurnal profiles of the parameters mentioned. The units corresponding to amplitude and MESOR are: fmoles/mg for IP_3_R and RyR binding assays; NADH/min/mg of protein for SERCA and PMCA activity; and normalized units for IP_3_R1-2, RyR and PMCA1-4 Western Blot (WB). * (*p* < 0.05) significant between AL vs RF (Student´s t-test). Not significant 24 h rhythmicity was designed as NS.

The RyR, the other important intracellular calcium-releasing channel in hepatic tissue, was also studied for ligand binding. According to a one-way ANOVA, [^3^H]-ryanodine binding showed differences among time points in both AL and RF groups (Figure 2E). Interestingly, there were no differences between these groups, as tested by a two-way ANOVA test, suggesting that the RF schedule did not affect the diurnal rhythmicity of the RyR. Clear differences in [^3^H]-ryanodine binding were observed in the RF group between ZT3 (before feeding) and ZT6 (after feeding) but not in the control groups (Fasted vs Refed) (Figure 2F). Hence, the RF group exhibited discrete modifications in the daily rhythm of [^3^H]-ryanodine binding (a phase advance of ~3 h, Table 2), but a significant effect at ZT3 in comparison to the Fasted group.

**Figure 3:**
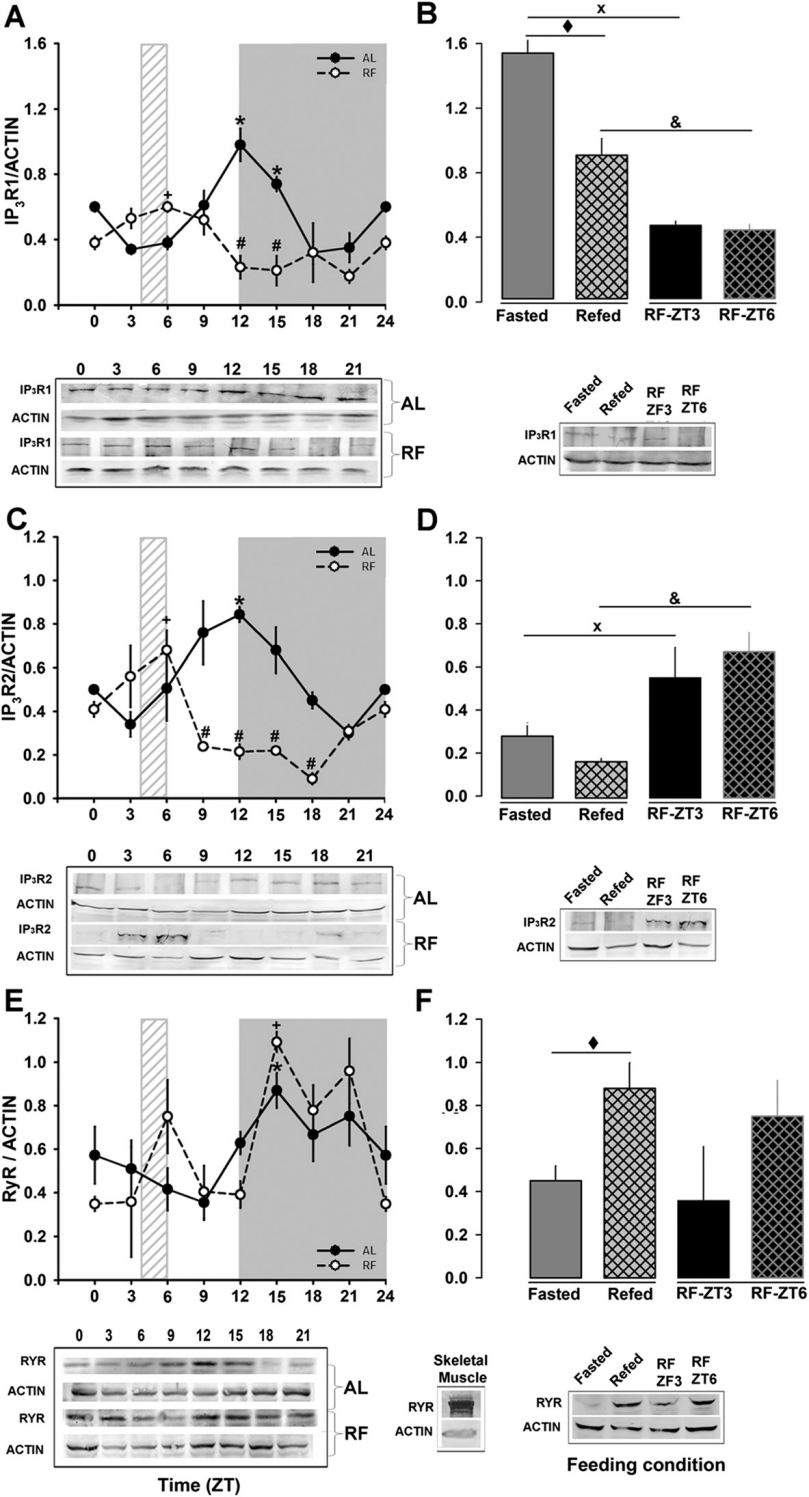
**Detection of hepatic IP_3_Rs and RyR.** The protein content of the calcium-release channels was evaluated by western blot (IP_3_Rs) and SDS-PAGE (RyR). Representative signals of IP_3_R1, IP_3_R2, and RyR and loading control (actin), and the daily rhythm profiles are shown in panels **A**, **C**, and **E**, respectively. Panels **B**, **D**, and **F** compare control groups of feeding condition (Fasted and Refed) for expression of the IP_3_R1, IP_3_R2, and RyR, respectively. Meanwhile IP_3_R1 and 2 were determined by conventional western blot, RyR protein was detected and quantified by stains-all methodology as described previously. Mean values for at least 4 independent experiments are shown. For IP_3_R type 1 and RyR proteins, 100 μg of the ER fraction was used. The plasma membrane fraction was used for IP_3_R type 2 (100 μg). Black circles correspond to AL and white circles to RF group. The light gray rectangle above x-axis indicates mealtime for the food restricted group (ZT4-ZT6). Mean values for at least 4 independent experiments are shown. Each data point was measured in triplicate. * (*p* < 0.05) significant difference between AL time points and **+** (*p* < 0.05) significant difference in the RF group between their time points (1-way ANOVA); # (*p* < 0.05) significant between AL vs RF (2-way ANOVA). ♦ (*p* < 0.05) significant difference between Fasted and Refed groups; x (*p* < 0.05) significant difference between Fasted and RF-ZT3; and & (*p* < 0.05) significant difference between Refed and RF-ZT6 (Student´s t-test).

To investigate if these changes were associated with fluctuating levels of receptor expression, IP_3_R and RyR were analyzed by Western blot. IP_3_R1 (the main isoform in the hepatic ER fraction) showed a pattern similar to the [^3^H]-IP_3_ binding assay: AL and RF displayed 24-h rhythmicity (one-way ANOVA, Figure 3A), and significant differences were found due to time and feeding condition in both groups (tested by two-way ANOVA). However, very different characteristics were observed in their daily rhythm: the AL group showed a clear peak at the beginning of the dark period (ZT12), whereas in the RF group the peak shifted 8 h toward the middle part of the light period (before and after RF schedule) and had a 50% lower amplitude (Table 2). Acute fasting promoted a significant increase in the IP_3_R1 expression that was reverted upon re-feeding (Figure 3B). This effect was not observed in the rats under RF, since the level of IP_3_R1 was similar before and after food access (RF at ZT3 and ZT6, Figure 3B). The PM fraction was examined for the presence of hepatic IP_3_R type 2. The level of liver IP_3_R2 protein showed daily rhythmicity in the AL and RF groups, but the amplitude was ~50% lower in the latter (Figure 3C); in addition, RF promoted a shift in the IP_3_R2 peak, from the transition between light and dark periods (ZT12) observed in AL rats, to the time of daytime feeding (ZT6) (Table 2), a phase advance of 6 h. Fasted and Refed controls also showed an evident decrease in comparison to the RF group at ZT3 and ZT6, respectively (Figure 3D). These results strongly suggest that the diurnal rhythms of hepatic IP_3_R1 and 2 are profoundly modified by RF (resulted from 3 weeks) but in a distinct way than in the Fasted and Refed groups, which resulted from an acute condition (1 day).

The other calcium-release channel located in liver microsomal membranes, the RyR, also showed a clear diurnal rhythm in the AL group, with a significant elevation of the RyR protein (≈40%) during the dark period (Figure 3E). RF promoted 2 main changes in this rhythm: 1) after feeding (ZT6), an increase (≈80%) was observed; 2) at the beginning of the dark period (ZT15) a second, even larger, peak was detected. In addition, after food access (ZT6), the RF group showed an increase (≈30%) in comparison to the group before meal time (ZT3) that resulted significant among the Fasted versus Refed group (Figure 3F).

### SERCA and PMCA: activity and protein expression during restricted feeding / FEO expression

**Figure 4:**
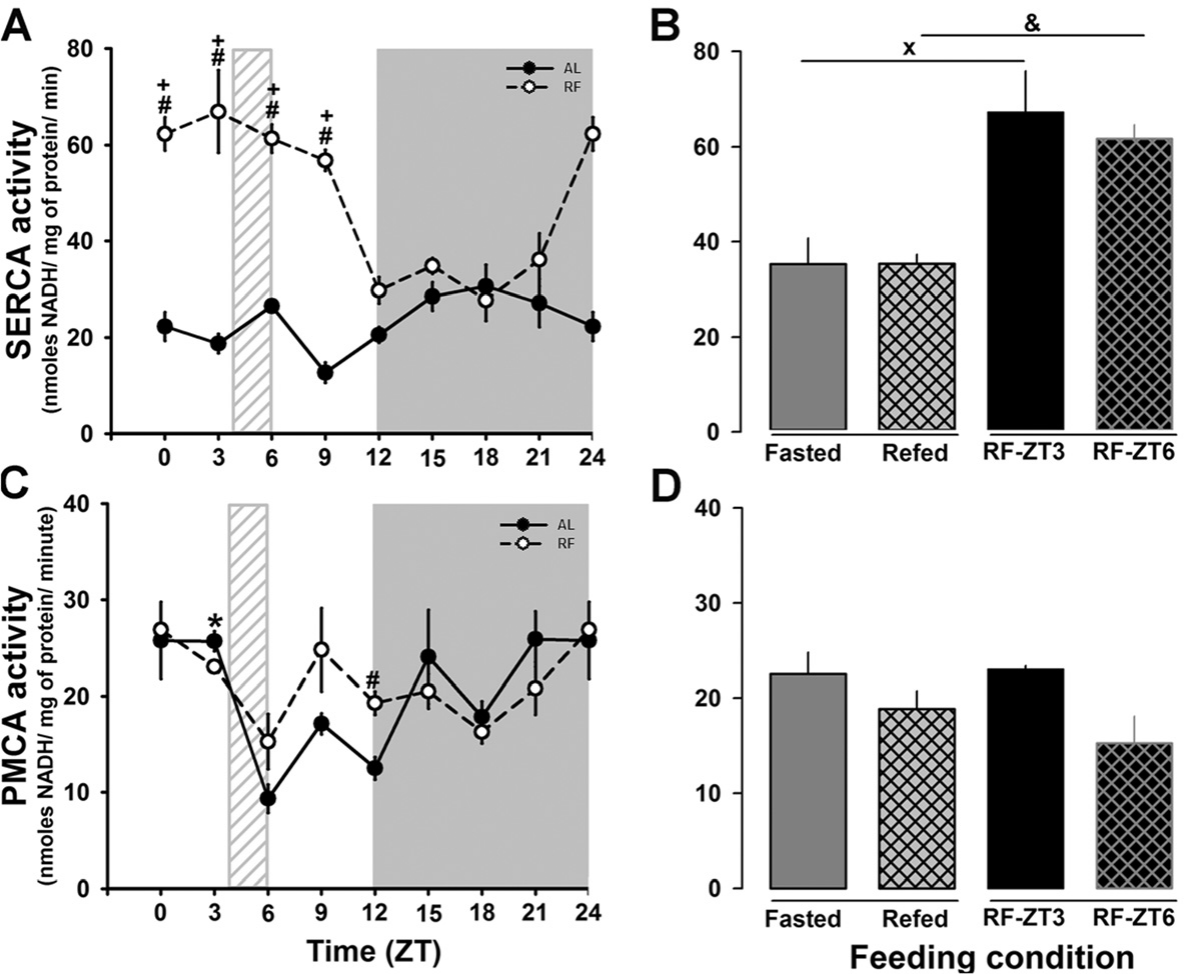
**Hepatic SERCA and PMCA activities Activity of Ca^2+^-ATPase in microsomal and plasma membrane fractions was measured as described in the methods.** The daily profile of SERCA and PMCA activities are shown in panel **A** and **C**, respectively. Black circles correspond to AL, and white circles to RF group. The light gray rectangle above x-axis indicates mealtime for the food restricted group (ZT4-ZT6). The corresponding comparisons between feeding conditions (Fasted and Refed) are showed in panel **B** for SERCA activity and in panel **D** for PMCA activity. Mean values of 4 independent experiments are shown. Each data point was measured in triplicate. **+** (*p* < 0.05) significant difference in the RF group between their time points (1-way ANOVA); # (*p* < 0.05) significant between AL vs RF (2-way ANOVA). x (*p* < 0.05) significant difference between Fasted and RF-ZT3 and & (*p* < 0.05) significant difference between Refed and RF-ZT6 (Student´s t-test).

SERCA activity in the AL group did not change over 24 h (Figure 4A). In contrast, RF rats showed a clear peak and an evident increase in the SERCA activity during the light phase (*P < 0.001*; one-way ANOVA, Figure 4A). Hence, significant rhythmicity was detected in the RF, but not in the AL group (Table 2). This clear difference associated with the restricted feeding protocol/FEO expression was even more remarkable since no changes were detected in feeding control rats (Fasted vs Refed). PMCA activity was reduced (≈50%) in the AL group at the end of the light period, whereas RF rats showed no daily pattern of PMCA activity (except for a reduction observed at ZT9-12) (Figure 4C); no daily rhythmicity was detected for these 2 groups. In addition, no change was observed in the feeding control groups (Figure 4D).

**Figure 5:**
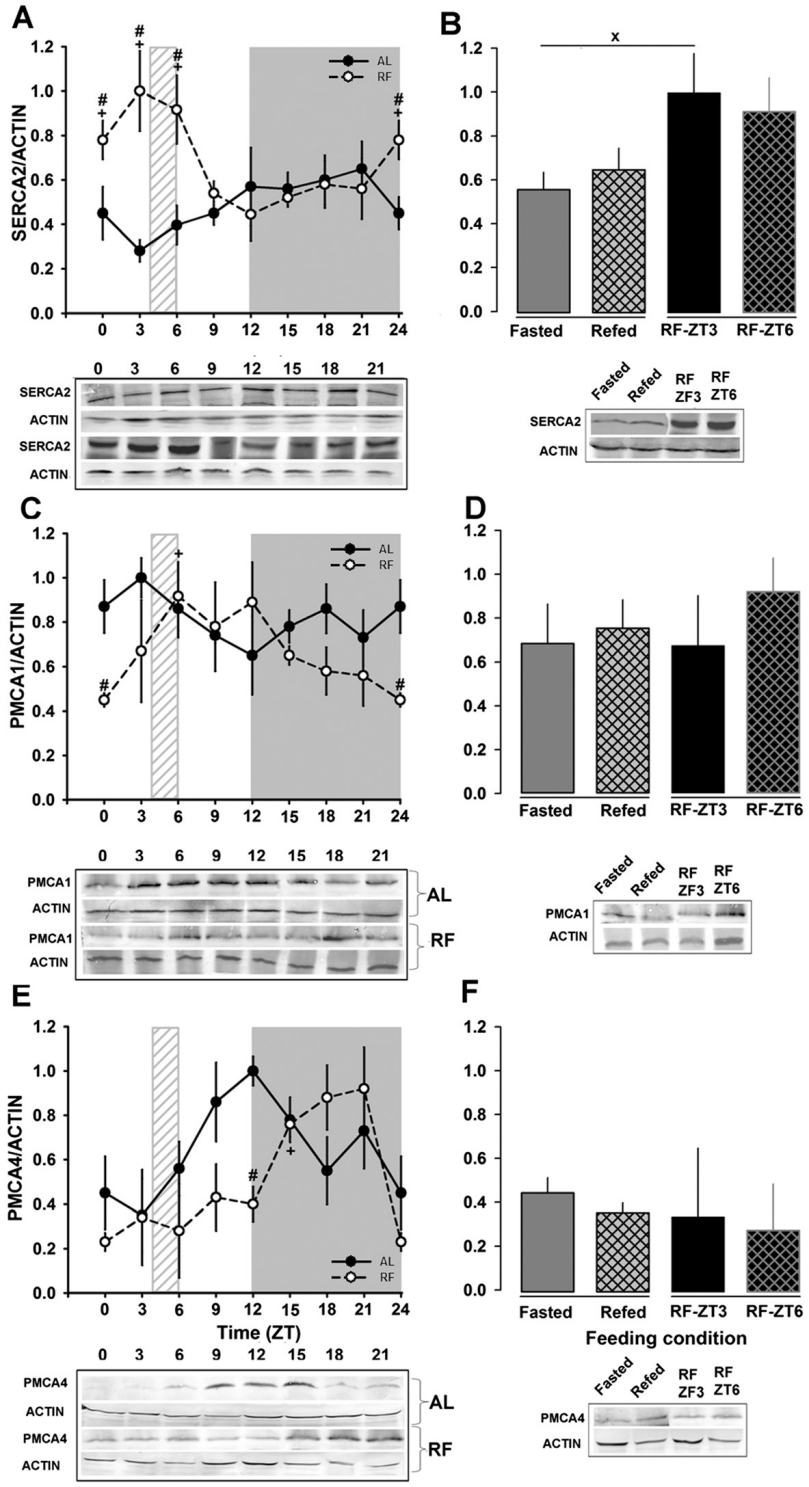
**Western blots of hepatic SERCA and PMCA.** The daily rhythm profiles and representative western blots of the Ca^2+^-ATPases SERCA2, PMCA1, and PMCA4 are shown in panels **A**, **C**, and **E**, respectively. Comparisons among the different feeding conditions are shown in panels **B**, **D**, and **F** for expression of SERCA2, PMCA1, and PMCA4, respectively. Mean values for at less 4 independent experiments are shown. For SERCA2 protein, 100 μg of ER fraction was used. The plasma membrane fraction (100 μg) was used in the case of PMCA type 1 and 4 proteins. Black circles correspond to AL, and white circles to RF group. The light gray rectangle above x-axis indicates mealtime for the food restricted group (ZT4-ZT6). Mean values for at least 4 independent experiments are shown. Each data point was measured in triplicate. **+** (*p* < 0.05) significant difference in the RF group between their time points (1-way ANOVA); # (*p* < 0.05) significant between AL vs RF (2-way ANOVA). x (*p* < 0.05) significant difference between Fasted and RF-ZT3 (Student´s t-test).

To complement the enzymatic measurements of SERCA and PMCA activity, the subcellular fractions were assayed for these proteins by Western blot. The results shown in Figure 5A confirmed that SERCA2 protein (the isoform expressed in liver) did not change significantly during the 24-h period in AL rats, even though ZT18 resulted higher than ZT3. The Cosinor test failed to detect a daily rhythm, but he RF group resulted rhythmic as in the case of the SERCA activity. This result was confirmed by the Cosinor test (Table 2). A significant difference (*P < 0.001*; Student t-test) between the Fasted and RF-ZT3 group was also observed (Figure 5B).

Liver expresses the PMCA isoforms 1 and 4 [25]. Since the enzymatic assay did not distinguish among PMCA isoforms, a Western blot for isoforms 1 and 4 was done. The PMCA1 protein did not oscillate in the AL group, whereas the RF group showed a significant daily rhythmicity (Figure 5C). The PMCA1 acrophase was at the end of the light period (Table 2). A similar pattern was seen for PMCA4: AL did not present a daily rhythmicity and RF groups showed significant differences with time (Figure 5E). Interestingly, the Acrophase of PMCA4 in the RF group was delayed by ~ 5 h (Table 2). However, the protein level of neither PMCA1 nor PMCA4 was affected by alimentary condition (Fasted vs Refed) (Figure 5D and F).

### Rhythmical analysis of the hepatic calcium handling channels and ATPases during restricted feeding / FEO expression

The cosinor test helps to determine a potential 24-h rhythm that is consistent with a sinusoidal curve. The rhythms for all the proteins studied in this project were confirmed first by a one-way ANOVA prior to the 24-h cosinor test (Table 1). Consistent with the corticosterone and Per1 levels measured in rats expressing the FEO (Figure 1), their acrophases were clearly shifted around the time of food access (ZT4 and ZT6) (Table 1). A 24-h rhythm was detected for IP_3_R types 1 and 2, RyR, and PMCA types 1 and 4, either with AL or RF protocols or with both AL and RF protocols (*P < 0.05*, Table 2).

### Effect of restricted feeding/FEO expression on the hepatic zonal distribution of calcium-handling proteins

**Figure 6:**
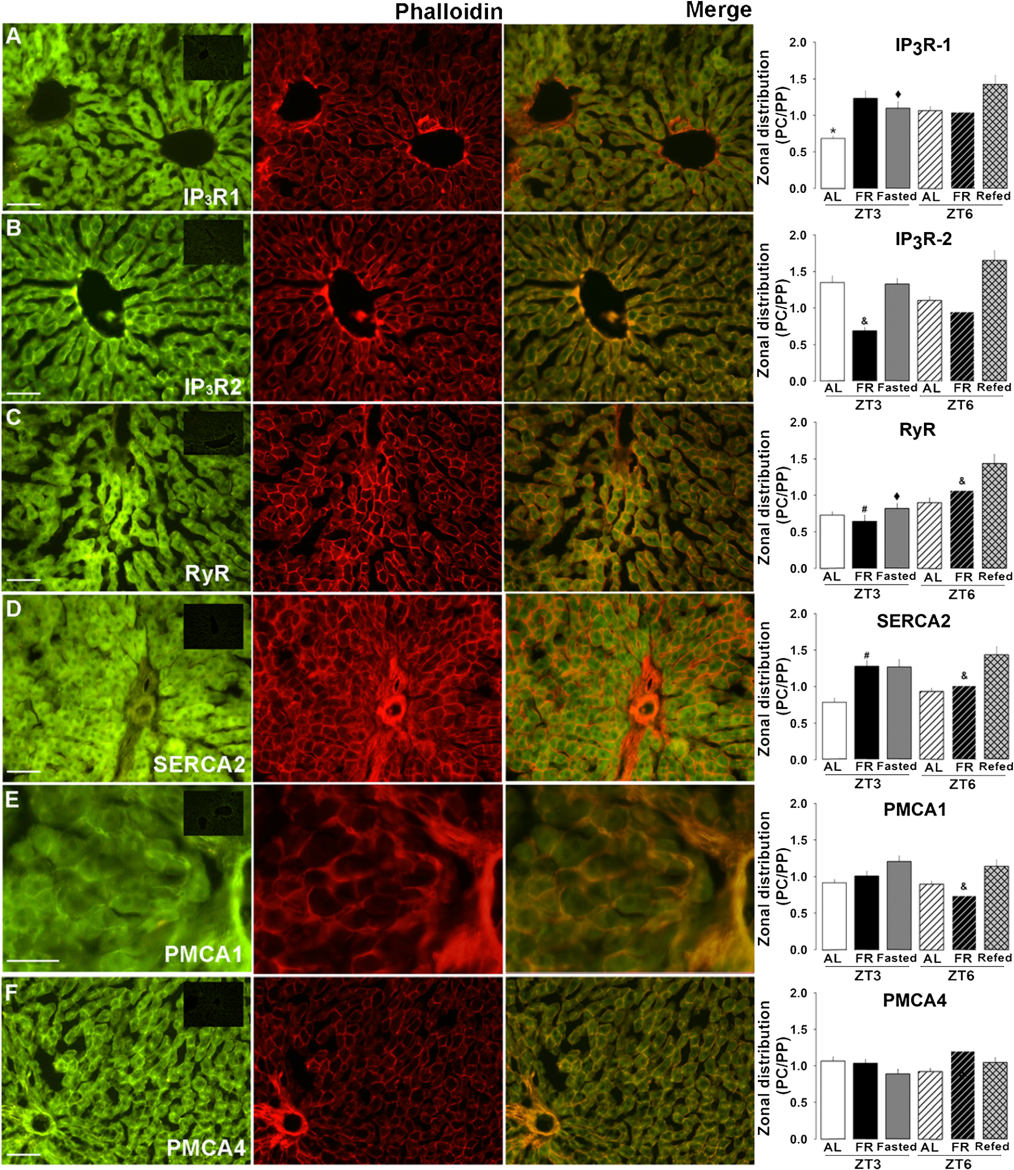
**Zonal distribution of hepatic calcium-handling proteins.** Zonal distribution of IP_3_R type 1 (panel **A**), IP_3_R type 2 (panel **B**), ryanodine receptor (panel **C**), SERCA type 2 (panel **D**), PMCA type 1 (panel **E**), and PMCA type 4 (panel **F**) are shown. A negative control (primary antibody omitted) is shown in the insert of panel **A**. In order to outline liver cells, rhodamine-conjugated phalloidin was used to detect actin associated to membrane as showed in the central images. Each figure shows semi-quantitative analysis of calcium-handling protein distribution (pericentral/periportal ratio) for each time (AL-ZT3, AL-ZT6, RF-ZT3, and RF-ZT6) and feeding condition (Fasted and Refed). * (*p* < 0.05) significant difference between AL time points (ZT3 vs ZT6); # (*p* < 0.05) AL vs RF; ♦ (*p* < 0.05) Fasted vs Refed groups and & (*p* < 0.05) Refed vs RF-ZT6 (Student´s t-test).

To compare the changes in the binding and activities of the hepatic calcium channels and ATPases during FEO expression in the specialized hepatocyte populations, we analyzed for the presence of these proteins within the hepatic acinus, distinguishing among the PP and PC zones (Figure 6). Since the main metabolic and physiological changes in the parameters studied were detected at ZT 3 (before food access in RF protocol) and ZT6 (after feeding in RF protocol), this analysis was done only at these times. The immunohistochemistry of IP_3_R1 (Figure 6A) showed a homogeneous distribution along the hepatic acinus except in the AL group at ZT3, which showed an increase in the periportal zone, and the Refed control group in which this protein was predominantly in the pericentral zone. The IP_3_R2 distribution in control rats did not show zonal heterogeneity; in contrast, for RF rats, it was higher in the periportal zone at ZT3 and ZT6 (Figure 6B). Again, the control Refed group showed an increase in IP_3_R2 in the pericentral zone in comparison to the RF rats at ZT6. The ryanodine receptor was found primarily in the periportal zone in almost all conditions (Figure 6C). RyR was detected in the pericentral zone only at ZT6 in RF rats, as well as in the Refed control group. SERCA2 showed a periportal distribution in the AL group, but not in the RF or control feeding groups (Figure 6D). PMCA1 occurred in the perportal zone in the RF group at ZT6, but was uniformly distributed throughout the hepatic acinus in all other groups (Figure 6E). PMCA4 distribution also showed no zonal preference, unlike the other calcium-handling proteins (Figure 6F).

## Discussion

Many reports indicate that food entrainment profoundly affects liver physiology, from gene expression studied by microarrays [12] to a variety of metabolic adaptations [11,13,14,17,18]. In this context, a report from Stokkan et al., [10] indicates that the peak of Per1, measured by bioluminescent luciferase activity, was modified to coincide with the time of food access. A similar result was reproduced in Figure 1, indicating a successful entrainment to mealtime in the RF group. A similar entrainment was also evident in the daily corticosterone daily profile, since the RF group showed 2 peaks (one corresponding to the FFA and the other to the light–dark transition), whereas the AL group showed only the second peak. This result suggests that even though the daytime food cue strongly modifies the timing system, the influence of the SCN is still present during the FEO expression.

A role for intracellular calcium dynamics in regulating the circadian clock has been reported in several models [47]. This secondary messenger is involved in the entrainment process [48] and the 24-h rhythmicity of clock genes [49] as well as in the components of the circadian output [50]. For example, RyR2 seems to participate in the clock machinery function of the SCN by modulating the membrane potential [51]. However, so far no reports exist regarding the daily rhythm characterization of the liver calcium-handling proteins in the protocol of daytime restricted feeding/FEO expression. A previous report, using a pharmacological approach in hepatic explants, showed that IP_3_R, RyR, and SERCA modulated the clock gene *Per1* rhythmicity [52].

This project demonstrated that the activity and protein levels of the hepatic calcium releasing channels IP_3_R and RyR and the calcium pump SERCA have a daily rhythm that, in addition, is modified by changes in meal access time. It is known that IP_3_R types 1 and 3 as well as the RyR type 2 oscillate with a circadian periodicity in the SCN [49,53]. In addition, IP_3_R has been suggested as an element that contributes to the SCN entrainment mechanism [49]. In liver tissue, the IP_3_R has an important role regulating the calcium oscillations involved in metabolic processes such as bile production, mitochondrial activity, and the gluconeogenic pathway [54,55]. It was reported that the period of the clock gene Per1 was lengthened by inhibiting the IP_3_R using 2-APB (2-Aminoethoxydiphenyl borate) in liver explants [52]. These data strongly suggest that calcium handled by the IP_3_R participates in regulating the hepatic molecular clock rhythmicity. The present project showed that the levels of hepatic IP_3_R protein and binding activity displayed robust daily rhythms in the AL group, but as a consequence of daytime food restriction, their daily fluctuations are modified. The changes in the properties of the IP_3_Rs were also observed in the control groups of feeding conditions (Fasted and Refed), which suggests that the IP_3_Rs are under circadian and metabolic control. In food restriction, the circadian control could be exerted by the SCN as well as by the FEO. The IP_3_R has been postulated to be a positive modulator of glyconeogenisis in the liver as an element responsive to glucagon stimulus [55]. Our group has previously reported daily fluctuations of glucagon in RF conditions that show good correspondence to the daily variations of IP_3_Rs observed in this report (both showed peaks in the activity and protein levels of IP_3_R at the time when animals are expecting their meal) [13]. In addition, our data of daily variations of IP_3_R properties during restricted feeding showed good coincidence with the 24-h changes observed in gluconeogenic enzymes (manuscript in preparation).

The hepatic RyR has been related to mitochondrial respiratory activity, glycogen catabolism, liver regeneration, and protection against hypoxic stress [27,56,57]. The isoform of RyR within the liver is truncated, but it still shows the pharmacological profile of the better-studied skeletal muscle RyR isoform [24]. At 1 mM, ryanodine inhibited RyR and lengthened the circadian period of Per1-luc expression in liver explants of rats feeding *ad libitum*[52]. In contrast, liver explants from animals under restricted meal schedule did not show modification in the PER1::LUC rhythmicity. Our data indicated a clear daily rhythm in RyR protein levels and ligand-binding properties in the AL group. Interestingly, the RF group showed similar patterns, which supports the finding in liver explants [52], and suggests that RyR is not affected by the RF protocol. These data seem to indicate that the role played by the RyR in the timing system of the liver differs from the one in the SCN [51].

It is well accepted that the calcium ATPases SERCA and PMCA function as enzymes that maintain calcium homeostasis by intruding this cation to the ER or extruding it to the extracellular space, respectively. Both calcium pumps modulate the frequency of [Ca^2+^] oscillation in response to hepatic endocrine stimuli [25]. When SERCA or PMCA are overexpressed, expression of the other decreases, suggesting a finely tuned communication between the two enzymes [58]. SERCA is also involved in the ER stress response as well as in regulating lipid metabolism. Overexpression of hepatic SERCA2 is associated with a significant rise in lipogenic activity, whereas at the same time, it mitigates ER stress in obese mice [59]. However, there are no reports studying daily fluctuations of these ATPases. One of the most remarkable results of this study is the large increases in SERCA protein and activity observed in the RF group before and after the feeding time (from ZT0 to ZT9), which is coincident with the onset of FAA (before meal) and the intense hyperphagic event (after mealtime). A possible interpretation of elevated SERCA activity during the restricted feeding/FEO expression is that high calcium within the ER lumen and the low cytoplasmic calcium could be needed in the reticular response in preparation for food processing and in the lipolytic response during nutrient handling [18].

**Figure 7:**
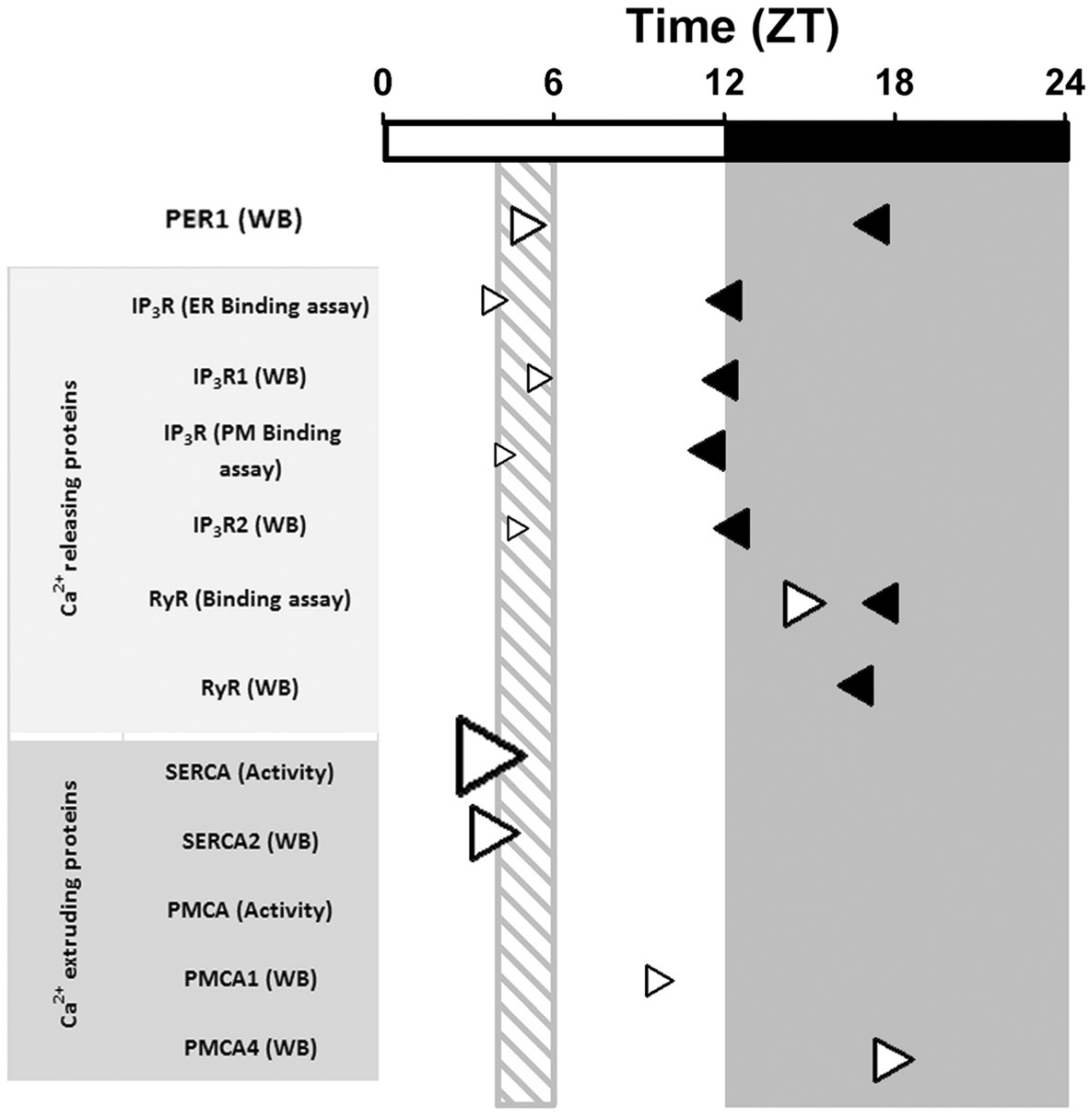
**Acrophase summary of hepatic calcium-handling protein daily rhythms.** Times of peak activity and protein levels for each of the studied proteins are shown for the AL group (black arrowheads) and for the RF group (white arrowheads). The dark gray rectangle indicates the dark period, and the ZT time is shown above. Each arrowhead from the RF group was normalized with respect to its control, and its size indicates the amplitude of the daily rhythm in comparison to the AL group. The dashed rectangle shows the time of food access for the RF group. Calcium-handling protein activity or expression levels that did not show 24-h rhythmicity were omitted.

Regarding the daily patterns characterized in the hepatic calcium-release channels and pumps, almost all the proteins that had 24-h rhythmicity, also showed a shift in their Acrophase (Figure 7). Only RyR peaks (activity and protein level) were not totally shifted to meal time in the RF protocol. The RyR binding assay demonstrated a modest 3 h phase advance in the RF group with respect to the AL group (Figure 2E, Table 2), whereas the other calcium-handling proteins showed phase advances with an average of 9 h (Figure 7, Table 2). Since amplitude is another rhythmic parameter that must be taken into account, it is noteworthy that SERCA activity amplitude in RF rats increased almost 4 fold in comparison to the AL group (Figure 4A, Table 2). MESOR was no exception, since changes in both IP_3_R isoforms (a 50% decrease) and SERCA activity (~2-fold increase) during RF protocol/FEO expression were detected. The temporal profile of PMCA activity did not show a 24-h rhythm according to cosinor for both the AL and RF group. However, an ultradian 8-h rhythm was detected for this calcium pump activity in the AL group (data not shown); meanwhile a 24-h period rhythm was observed for both PMCA isoforms protein levels in the case of the RF group (Table 2).

Although IP_3_R2 zonal distribution within the hepatic acinus has been described [27], those for RyR, SERCA, and PMCA were studied for the first time, including the possible influence by feeding or chronobiological factors. Using a protocol of liver regeneration, IP_3_R type 2 as well as hormone receptors responsible for IP_3_R activation (glucagon and adrenaline receptors) were detected mainly in PP hepatocytes [20,27]. A significant PC distribution was seen for adrenergic receptors [41]. In addition, it was tested that hepatic calcium waves induced by the activation of the vassopresin receptor start in the PP and then disperse towards the PC zone [47]. Circadian variation of glycogen deposition was reported by histological studies for both zones. The proteins studied in our project differed in amount and activity between fasted and ZT3 or between re-fed and ZT6, even though they are under similar feeding conditions; this can be explained in the context in which FEO expression (induced by the RF condition) produces characteristic rheostatic adaptations in liver physiology [14]. This could be related with glycogen breakdown, we found that glycogen is more abundant at ZT3 than after a single 21-h fast, which confirms that circadian meal entrainment establishes a new physiological state in hepatic tissue [15].

Even though our data did not show a direct link between modifications in calcium-handling proteins and intracellular calcium dynamics associated with food entrainment, our results indicate a clear chronostatic adaptation for the liver calcium channels and ATPases considered in this study. Some authors have suggested a possible role of CamKII (Calmodulin Kinase II) as mediator of the coupling between intracellular calcium and circadian oscillations [60]. More experiments are needed to define if the calcium-handling proteins have an influence on: 1) the molecular clock of the liver, 2) the synchronization process, or 3) the output response of the liver oscillator.

As summarized in Figure 7, the activity, presence, and zonal distribution of liver IP_3_Rs, RyR, SERCA, and PMCA were modified differentially in ad libitum and daytime restricted feeding protocols. Whereas IP_3_R and SERCA changed during preprandial time, RyR and PMCA modifications were largely postprandial. The phase of the peaks in the daily rhythms of IP_3_Rs, RyR, and PMCA1 in the AL group was coincident with the active time of the rats, whereas the temporal pattern of activity and protein levels of IP_3_Rs, SERCA, and PMCA1-4 changed toward the meal access schedule in the animals under RF. These data suggest that during the reosthatic or allostatic adaptations shown by the liver associated with FEO expression, there exists a differential coordination of these calcium-handling elements which could have a direct impact on hepatic physiology and metabolism.

## Conclusion

Our results indicate that a daily rhythmic regulation occurs in the calcium-handling proteins, and very likely in hepatic calcium signaling. The RF condition promotes an adjustment in the activity, protein level, and zonal distribution of these calcium channels and ATPases (Figure 7). Hence, these results demonstrate that important elements in the intracellular calcium dynamics of the liver exhibit daily variations in the control condition of ad libitum feeding. This rhythmicity is further modulated during a protocol of daytime RF and the concomitant FEO expression.

## Competing interests

The authors declare that they have no conflicts of interest, financial or otherwise.

## Authors' contributions

AB-R, KC-G and OV-M performed experiments; AB-R, RA-R and MD-M analyzed data; AB-R prepared figures; AB-R, RA-R and MD-M conceived and designed the research; AB-R, RA-R and MD-M interpreted the results; AB-R and MD-M edited and revised manuscript. All authors approved read and approved the final manuscript.

## References

[B1] Schwartz MW Woods SC Porte D Jr Seeley RJ Baskin DG Central nervous system control of food intake Nature 2000 406 661 671 10766253 10.1038/35007534

[B2] Mistlberger RE Circadian food anticipatory activity: formal models and physiological mechanisms Neurosci Biobehav Rev 1994 18 171 195 10.1016/0149-7634(94)90023-X 8058212

[B3] Mendoza J Circadian clocks: setting time by food J Neuroendocrinol 2006 19 127 137 17214875 10.1111/j.1365-2826.2006.01510.x

[B4] Marchant EG Mistlberger RE Anticipation and entrainment to feeding time in intact and SCN-ablated C57BL/6j mice Brain Res 1997 765 273 282 10.1016/S0006-8993(97)00571-4 9313900

[B5] Novin D Is there a role for the liver in the control of food intake? Am J Clin Nutr 1985 42 1050 1062 4061358 10.1093/ajcn/42.5.1050

[B6] Langhans W Role of the liver in the control of eating: what we know – and what we do not know Neurosci Biobehav Rev 1996 20 145 153 10.1016/0149-7634(95)00045-G 8622821

[B7] Friedman MI Horn CC Ji H Peripheral signals in the control of feeding behavior Chem Senses 2005 30 i182 i183 10.1093/chemse/bjh174 15738103

[B8] Rawson NE Ji H Friedman MI 2,5-D-mannitol increases hepatocyte calcium: implications for a hepatic hunger stimulus Biochem Biophys Acta 2003 1642 59 66 10.1016/S0167-4889(03)00099-5 12972294

[B9] Ji H Friedman MI Compensatory hyperphagia after fasting tracks recovery of liver energy status Physiol Behav 1999 68 181 186 10.1016/S0031-9384(99)00173-0 10627079

[B10] Stokkan KA Yamazaki S Tei H Sakaki Y Menaker M Entrainment of the circadian clock in the liver by feeding Science 2001 291 490 493 10.1126/science.291.5503.490 11161204

[B11] Davidson AJ Castañon C Stephan FK Daily oscillations in liver function: diurnal vs circadian rhythmicity Liver Int 2004 24 179 186 10.1111/j.1478-3231.2004.00917.x 15189266

[B12] Báez-Ruiz GA Vázquez-Martínez O Ramírez J Díaz-Muñoz M The food entrainable oscillator studied by DNA microarray: what is the liver doing during food anticipatory activity? Biol Rhythm Res 2005 36 83 97 10.1080/09291010400028674

[B13] Díaz-Muñoz M Vázquez-Martínez O Aguilar-Roblero R Escobar C Anticipatory changes of liver metabolism and entrainment of insulin, glucagon and corticosterone in food-restricted rats Am J Physiol Regul Integr Comp Physiol 2000 279 R48 R56 10.1152/ajpregu.2000.279.6.R2048 11080068

[B14] Báez-Ruiz GA Vázquez-Martínez O Escobar C Aguilar-Roblero R Díaz-Muñoz M Metabolic adaptation or liver mitochondria during restricted feeding schedules Am J Physiol Gastrointest Liver Physiol 2005 289 G1015 G1023 10.1152/ajpgi.00488.2004 15976385

[B15] Díaz-Muñoz M Vázquez-Martínez O Báez-Ruiz A Martínez-Cabrera G Soto-Abraham MV Avila-Casado MC Larriva-Sahd J Daytime food restriction alters liver glycogen, tryacylglycerols, and cell size: a histochemical, morphometric, and ultrastructural study Comp Hepatol 2010 9 5 10.1186/1476-5926-9-5 20178596 PMC2838809

[B16] Escobar C Díaz-Muñoz M Encinas F Aguilar-Roblero R Persistence of metabolic rhythmicity during fasting and its entrainment by restricted feeding schedules in rats Am J Physiol Regul Integr Comp Physiol 1998 274 R1309 R1316 10.1152/ajpregu.1998.274.5.R1309 9644044

[B17] Martínez-Merlos MT Ángeles-Castellanos M Díaz-Muñoz M Aguilar-Roblero R Mendoza J Escobar C Dissociation between adipose tissue signals, behavior and the food-entrained oscillator J Endocrinol 2004 181 53 63 10.1677/joe.0.1810053 15072566

[B18] Rivera-Zavala JB Báez-Ruiz A Díaz-Muñoz M Changes in the 24 h rhythmicity of liver PPARs and peroxisomal markers when feeding is restricted to two daytime hours PPAR Res 2011 2011 261584 doi:10.1155/2011/261584 21822420 10.1155/2011/261584 PMC3092493

[B19] McEwen B Physiology and neurobiology of stress and adaptation: central role of the brain Physiol Rev 2007 87 873 904 10.1152/physrev.00041.2006 17615391

[B20] Gaspers LD Thomas AP Calcium signaling in the liver Cell Calcium 2005 38 329 342 10.1016/j.ceca.2005.06.009 16139354

[B21] Berridge MJ Bootman MD Roderick HL Calcium signalings: dynamics, homeostasis and remodeling Nature Mol Cell Biol 2003 4 517 529 10.1038/nrm1155 12838335

[B22] Hirata K Pusl T O´Neill AF Dranoff D Nathanson MH **The type II Inositol 1,4,5-Trisphosphate Receptor can trigger Ca**^**2+ **^**waves in rat hepatocytes** Gastroenterol 2002 122 1088 1100 10.1053/gast.2002.32363 11910359

[B23] Nagata J Guerra MT Shugrue CA Gomes DA Nagata N Nathanson MH Lipid rafts establish calcium waves in hepatocytes Gastroenterology 2007 133 256 267 10.1053/j.gastro.2007.03.115 17631147 PMC2825880

[B24] Pierobon N Renard-Rooney DC Gaspers LD Thomas AP Ryanodine receptor in the liver J Biol Chem 2006 10 34086 34095 16973607 10.1074/jbc.M607788200

[B25] Delgado-Coello B Trejo R Mas-Oliva J Is there a specific role for the plasma membrana Ca^2+^-ATPase in the hepatocytes? Mol Cell Biochem 2006 30 1 15 16477375 10.1007/s11010-005-9060-z

[B26] Jungermann K Kietzmann T Zonation of parenchymal and nonparenchymal metabolism in liver Annu Rev Nutr 1996 16 179 203 10.1146/annurev.nu.16.070196.001143 8839925

[B27] Nicou A Serriere V Hilly M Prigent S Combettes L Guillon G Tordjmann T Remodelling of calcium signaling during liver regeneration in the rat J Hepatol 2007 46 247 256 10.1016/j.jhep.2006.08.014 17125880

[B28] Gasbarrini A Borle AB Caraceni P Colantoni A Farghali H Trevisani F Bernardi M van Thiel DH Effectof ethanol on adenosine trisphosphate, cytosolic free calcium, and cell injury in rat hepatocytes: time course and effect of nutritional status Dig Dis Sci 1996 41 2204 2212 10.1007/BF02071401 8943973

[B29] Portaluppi F Smolensky MH Touitou Y Ethics and methods for biological rhythm research on animals and human beings Chronobiol Int 2010 25 1911 1929 20969531 10.3109/07420528.2010.516381

[B30] Díaz-Muñoz M Cañedo-Merino R Gutiérrez-Salinas J Hernández-Muñoz R Modifications of intracellular calcium release channels and calcium mobilization following 70% hepatectomy Arch Biochem Biophys 1998 349 105 112 10.1006/abbi.1997.0396 9439588

[B31] Janicki PK Wise PE Belous AE Pinson CW Interspecies differences in hepatic Ca^2+^-ATPase activity and the effect of cold preservation on porcine liver Ca^2+^-ATPase function Liver Transpl 2001 7 132 139 10.1053/jlts.2001.21459 11172397

[B32] Damiani E Tobaldin G Volpe P Margreth A Quantitation of ryanodine receptor of rabbit skeletal muscle, heart and brain Biochem Biophys Res Commun 1991 175 858 865 10.1016/0006-291X(91)91644-R 2025259

[B33] Lowry OH Rosebrough NJ Farr AL Randall RJ Protein measurement with the folin phenol reagent J Biol Chem 1951 193 265 275 14907713

[B34] Harper AE Bergmeyer H Glucose-6-phosphate activity measurement Methods of enzymatic analysis 1965 New York: Academic Press 788 792

[B35] Widnell CC Unkeless JC Partial purification of a lipoprotein with 5′ nucleotidase activity from membranes of rat liver cells Proc Natl Acad Sci USA 2004 61 1050 1057 5246541 10.1073/pnas.61.3.1050 PMC305434

[B36] Furiuchi T Simon C Fujino I Yamada N Hagesawa M Miyawaki A Yoshikawa S Guenét JL Mikoshiba K Widespread expression of inositol 1,4,5-trisphosphate receptor type 1 gene (Insp_3_R1) in the mouse central nervous system Receptors Channels 1993 1 11 24 8081710

[B37] Fricke U Tritosol: a new scintillation cocktail based on triton X-100 Anal Biochem 1975 63 555 558 10.1016/0003-2697(75)90379-6 1122028

[B38] Hamilton SL Mejía-Alvarez R Fill M Hawkes MJ Brush KL Schilling WP Stefani E [^3^H]PN200-110 and [^3^H]ryanodine binding and reconstitution of ion channel activity with skeletal muscle membranes Anal Biochem 1989 183 31 41 10.1016/0003-2697(89)90167-X 2559627

[B39] Saborido A Salgado J Megías A Measurement of sarcoplasmic reticulum Ca^2+^-ATPase activity and E-type Mg^2+^-ATPase activity in rat heart-homogenates Anal Biochem 1999 268 79 88 10.1006/abio.1998.3043 10036165

[B40] Laemmli UK Cleavage of structural proteins during the assembly of the head of bacteriophage T4 Nature 1970 227 680 685 10.1038/227680a0 5432063

[B41] Clair C Tran D Boucherie S Claret M Tordlmann T Combettes L Hormone receptor gradients supporting directional Ca^2+^ signals: direct evidence in rat hepatocytes J Hepatol 2003 39 489 495 10.1016/S0168-8278(03)00289-7 12971956

[B42] Hirata K Dufour JF Shibao K Knickelbein R O´Neill AF Bode HP Cassio D St-Pierre MV LaRusso NF Leite MF Nathanson MH Regulation of Ca^2+^ signaling in rat bile duct epithelia by Inositol 1,4,5-trisphosphate receptor isoforms Hepatol 2002 36 284 296 10.1053/jhep.2002.34432 PMC2987686 12143036

[B43] Lahm A Uhl M Lehr HA Ihling C Kreuz PC Haberstroch J Photoshop-based image analysis of canine articular cartilage after subchondral damage Arch Orthop Trauma Surg 2004 124 431 436 15365719 10.1007/s00402-004-0701-6

[B44] Caldelas I Tejadilla D González B Montúfar R Hudson R Diurnal pattern of clock gene expression in the hypothalamus of the newborn rabbit Neurosci 2007 144 395 401 10.1016/j.neuroscience.2006.09.020 17055660

[B45] Le Minh N Damiols F Tronche F Shütz G Schibler U Glucocorticoid hormones inhibit food-induced phase-shifting of peripheral circadian oscillators EMBO J 2001 20 7128 7136 10.1093/emboj/20.24.7128 11742989 PMC125339

[B46] Luna-Moreno D Vázquez-Martínez O Báez-Ruiz A Ramírez J Díaz-Muñoz M Food restricted schedules promote differential lipoperoxidative activity in rat hepatic subcellular fractions Comp Biochem Physiol A Mol Integr Physiol 2007 146 632 643 10.1016/j.cbpa.2006.02.039 16725359

[B47] Lambert CM Weaver DR Peripheral gene expression rhythms in a diurnal rodent J Biol Rhythms 2006 21 77 79 10.1177/0748730405281843 16461987

[B48] Edmunds LN Carré IA Tamponnet C Tong J The role of ions and second messengers in circadian clock function Chronobiol Int 1992 3 180 200 1319285 10.3109/07420529209064529

[B49] Hamada T Liou SY Fukushima T Maruyama T Watanabe S Mikoshiba K Ishida N The role of inositol trisphosphate-induced Ca^2+^ release from IP_3_ receptor in the rat suprachiasmatic nucleus on circadian entrainment mechanism Neurosci Lett 1999 263 125 128 10.1016/S0304-3940(99)00111-1 10213151

[B50] Lundkvist GB Kwak Y Hajime T Block G A calcium flux is required for circadian rhythm generation in mammalian pacemaker neurons J Neurosci 2005 25 7682 7686 10.1523/JNEUROSCI.2211-05.2005 16107654 PMC6725395

[B51] Aguilar-Roblero R Mercado C Alamilla J Laville A Díaz-Muñoz M Ryanodine receptor Ca^2+^ release channels are an output pathway for the circadian clock in the rat suprachiasmatic nuclei Eur J Neurosci 2007 26 575 582 10.1111/j.1460-9568.2007.05679.x 17686038

[B52] Báez-Ruiz A Díaz-Muñoz M Chronic inhibition of endoplasmic reticulum calcium-release channels and calcium-ATPase lenghtens the period of hepatic clock gene Per1 J Circadian Rhythms 2011 8 9 6 21740569 10.1186/1740-3391-9-6 PMC3142245

[B53] Díaz-Muñoz M Dent M Granados-Fuentes D Hall AC Hernández-Cruz A Harrington M Aguilar-Roblero R Circadian modulation of the Ryanodine receptor type 2 in the SCN of rodents Neuroreport 1999 10 481 486 10.1097/00001756-199902250-00007 10208575

[B54] Kruglov EA Gautam S Guerra MT Nathanson MH Type 2 inositol 1,4,5-trisphosphate receptor modulates bile salt export pump activity in rat hepatocytes Hepatol 2011 54 1790 1799 10.1002/hep.24548 PMC3205211 21748767

[B55] Wang Y Li G Goode J Paz JC Ouyang K Screaton R Fischer WH Chen J Tabas I Montminy M Inositol-1,4,5-trisphosphate receptor regulates hepatic gluconeogenesis in fasting and diabetes Nature 2012 485 128 132 10.1038/nature10988 22495310 PMC3343222

[B56] Komazaki S Ikemoto T Takeshima H Iino M Endo M Nakamura H Morphological abnormalities of adrenal gland and hypertrophy of liver in mutant mice lacking ryanodine receptors Cell Tissues Res 1998 294 467 473 10.1007/s004410051198 9799464

[B57] López-Neblina F Toledo-Pereyra LH Toledo AH Walsh J Ryanodine receptor antagonism protects the ischemic liver and modulates TNF-α and IL-10 J Surg Res 2007 140 121 128 10.1016/j.jss.2006.12.003 17359999

[B58] Kou TH Liu BF Yu Y Wuytack F Raeymaekers L Tsang W Coordinated regulation of the plasma membrane calcium pump and the sarco(endo)plasmic reticular calcium pump gene expression by Ca^2+^ Cell Calcium 1997 21 399 408 10.1016/S0143-4160(97)90051-8 9223676

[B59] Park SW Zhou Y Lee J Ozcan U Sarco(endo)plasmic reticulum Ca2 + −ATPase 2b is a major regulator of endoplasmic reticulum stress and glucose homeostasis in obesity Proc Natl Acad Sci USA 2010 107 19320 19325 10.1073/pnas.1012044107 20974941 PMC2984194

[B60] Harrisingh MC Nitabach MN Integrating circadian timekeeping with cellular physiology Cell 2008 320 879 880 10.1126/science.1158619 18487177

